# Horizontal transfer of a ß-1,6-glucanase gene from an ancestral species of fungal endophyte to a cool-season grass host

**DOI:** 10.1038/s41598-017-07886-2

**Published:** 2017-08-22

**Authors:** Hiroshi Shinozuka, Inoka K. Hettiarachchige, Maiko Shinozuka, Noel O. I. Cogan, German C. Spangenberg, Benjamin G. Cocks, John W. Forster, Timothy I. Sawbridge

**Affiliations:** 1 Agriculture Victoria, AgriBio, Centre for AgriBioscience, 5 Ring Road, La Trobe University, Bundoora, Victoria 3083 Australia; 2 Dairy Futures Cooperative Research Centre, Bundoora, Australia; 3 School of Applied Systems Biology, La Trobe University, Bundoora, Victoria 3086 Australia

## Abstract

Molecular characterisation has convincingly demonstrated some types of horizontal gene transfer in eukaryotes, but nuclear gene transfer between distantly related eukaryotic groups appears to have been rare. For angiosperms (flowering plants), nuclear gene transfer events identified to date have been confined to genes originating from prokaryotes or other plant species. In this report, evidence for ancient horizontal transfer of a fungal nuclear gene, encoding a ß-1,6-glucanase enzyme for fungal cell wall degradation, into an angiosperm lineage is presented for the first time. The gene was identified from *de novo* sequencing and assembly of the genome and transcriptome of perennial ryegrass, a cool-season grass species. Molecular analysis confirmed the presence of the complete gene in the genome of perennial ryegrass. No corresponding sequence was found in other plant species, apart from members of the Poeae sub-tribes Loliinae and Dactylidinae. Evidence suggests that a common ancestor of the two sub-tribes acquired the gene from a species ancestral to contemporary grass-associated fungal endophytes around 9–13 million years ago. This first report of horizontal transfer of a nuclear gene from a taxonomically distant eukaryote to modern flowering plants provides evidence for a novel adaptation mechanism in angiosperms.

## Introduction

The Poeae tribe of the Poaceae family is composed of a range of cool-season turf and forage grass species, including those of sub-tribes Loliinae and Dactylidinae^1^. Perennial ryegrass (*Lolium perenne* L.; sub-tribe Loliinae) is one of the most important pasture crop species for the dairy industry, and it has consequently been a primary target for improvement using molecular biology and genetic technologies^2^. Asexual fungal endophyte species of the genus *Epichloë* (syn. *Neotyphodium*) are symbionts of species belonging to the Poeae tribe genera *Lolium* and *Festuca*, and understanding of the symbiotic relationship is essential for improvement of the productivity of both forage and turf crops^3^. The fungal endophyte species rely on the plant host for nutrition, reproduction, and protection from abiotic and biotic stress. Benefits to the host plant include enhanced competitive abilities, tolerance to pathogens, and resistance to animal and insect herbivory^4^. Due to its agronomic importance, the molecular basis of the symbiosis has been the subject of detailed investigation, and deterrence of insect herbivory is largely due to the production of bioactive alkaloids, as well as *makes caterpillars floppy-like* (*mcf-like*) gene products^3, 5, 6^.

Horizontal gene transfer (HGT) has been an important source of evolutionary novelty in both prokaryotes and eukaryotes^7–9^. In flowering plant species, organelle genomes have served as both donors and recipients of gene transfer events^10^. The group I intron in the plant mitochondrial DNA gene, *cox1*, was acquired from a fungal species through an ancient transfer event^11^. The intron sequence of the mitochondrial genome has been found in a wide range of angiosperms from over 200 taxonomic families, and it is therefore, likely that the sequence was spread between taxa by plant-to-plant horizontal transfer, following the primary cross-kingdom transfer event^12, 13^. In Amborella (*Amborella trichopoda* Baill.), evidence for extensive ancestral horizontal transfer of mitochondrial DNA genes have been identified^14^. A phylogenetic approach revealed that the Amborella mitochondrial genome has acquired genes from mitochondria of green alga, moss and angiosperms. Due to the scale of gene integration, those foreign genes may have been transferred into Amborella through ‘mitochondria fusion’^14^. Horizontal transfer events of organelle genes (or sequences) may also be frequently observed between parasitic and host plants^15^, and be facilitated by the close and frequent interactions characteristic of such relationships.

In contrast to organellar genes, transfer of nuclear genes to angiosperms appears to have been rare^8–10^, and has been confined to date to genes originating from prokaryotes or other plant species such as green algae, mosses and other angiosperms^9, 15^. A previous systematic study, from investigation of four completely sequenced angiosperm genomes (those of *Arabidopsis thaliana* L., rice [*Oryza sativa* L.], sorghum [*Sorghum bicolor* L.], and poplar [*Populus trichocarpa* Torr. & A.Gray ex. Hook.]), found no evidence for HGT from fungal species, despite two and three highly reliable events for moss and lycophyte lineages, respectively^16^. It was consequently concluded that gene transfer from fungi into angiosperms must be exceedingly infrequent. In the current study, evidence for ancient horizontal transfer of a fungal ß-1,6-glucanase gene into an angiosperm lineage is presented. The ß-1,6-glucanase has an enzymatic activity for degradation of ß-1,6-glucan, which is commonly found in cell walls of fungi. The ß-1,6-glucanase genes have been isolated from fungal species, such as *Epichloë festucae* Leuchtm., Schardl and M.R. Siegel (the sexual counterpart to the perennial ryegrass endophyte), *Hypocrea lixii* Pat., and *Trichoderma harzianum* Rifai, and the gene is considered to be specific to fungi^17, 18^. This first report of horizontal transfer of a nuclear gene from a taxonomically distant eukaryote to modern flowering plants provides evidence for a novel adaptation mechanism in angiosperms.

## Results

### Identification of a putative plant ß-1,6-glucanase gene

**Figure 1:**
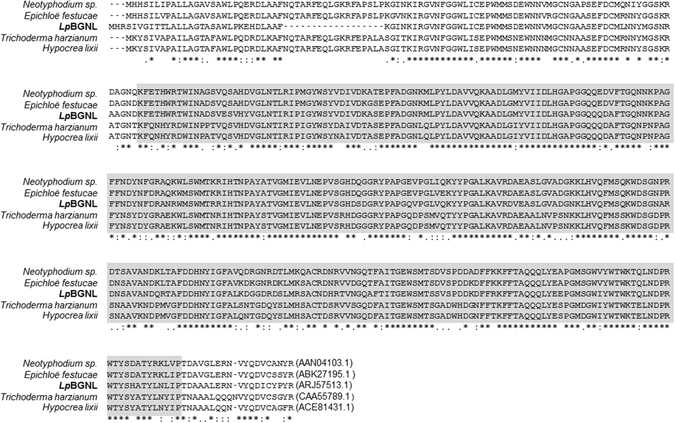
Amino acid sequence alignment of *Lp*BGNL and fungi ß-1,6-glucanase gene products. The aryl-phospho-beta-D-glucosidase domain is highlighted in grey. Dash (−) in the amino acid sequences shows a gap. Following the CLUSTAL W format, asterisk (*), colon (:) and dot (.) under the alignment denote ‘conserved amino acid residues’, ‘including conserved substitution(s)’ and ‘including semi-conserved substitution(s)’. NCBI UI is shown at the end of each sequence.

A single genotype of perennial ryegrass (Impact_04_) was subjected to whole-genome shotgun and transcriptome sequencing using the Illumina HiSeq platform^19^ (NCBI BioProject Accession: PRJNA379202). *De novo* assembly of sequencing reads generated a 7.2 kb genomic DNA sequence contig [NCBI GenBank unique identifier (UI): KY771173], which contained a putative ß-1,6-glucanase gene. The gene-like sequence was designated *Lp*BGNL (*Lolium perenne* ß-1,6-Glucanase-Like). *Lp*BGNL showed 74–90% and 72–82% identity at the DNA and amino acid sequence levels, respectively, to ß-1,6-glucanase genes of fungal taxa, such as *E*. *festucae*, *H*. *lixii*, and *T*. *harzianum* (Fig. 1 and Supplementary Table S1). No matching sequence, however, was identified in the full genome sequences of plants such as *A*. *thaliana*, rice, *Brachypodium distachyon* (L.) P. Beauv., barley (*Hordeum vulgare* L.) or wheat (*Triticum aestivum* L.), based on database searches.

### Genomic and genetic characterisation of LpBGNL

**Figure 2:**
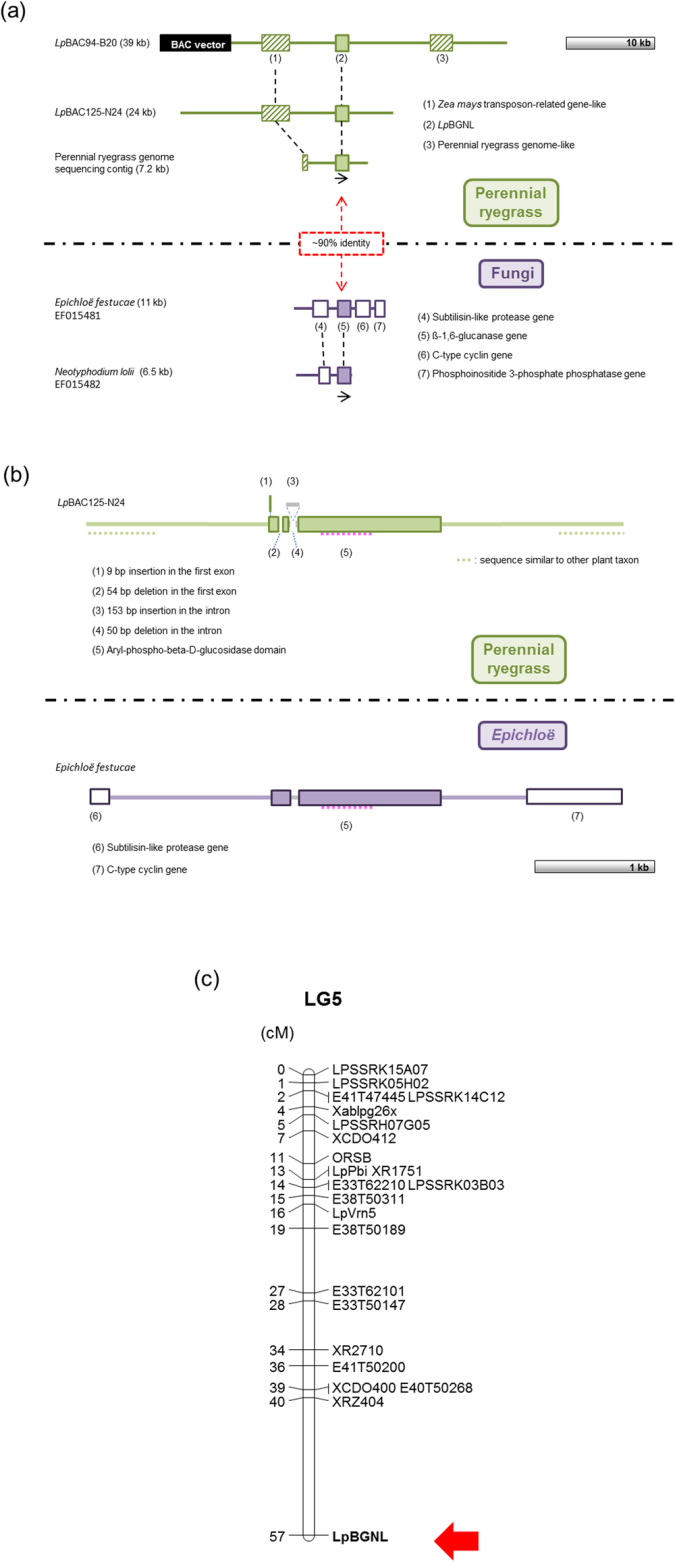
Genome structure of the *Lp*BGNL and *E*. *festucae* ß-1,6-glucanase genes and genetic linkage analysis for *Lp*BGNL. (**a**) Alignment of genome sequences from perennial ryegrass and *Epichloë* species. The light green-filled and striped boxes show the location of *Lp*BGNL and the plant genome-related sequence, respectively. The black-filled box represents the BAC vector. The purple-filled and empty boxes show the location of the ß-1,6-glucanase gene and flanking gene, respectively. The transcription direction of the genes is indicated with the arrow. Corresponding gene sequences are connected with black dashed lines. (**b**) Alignment of coding regions of the *Lp*BGNL and *Epichloë* ß-1,6-glucanase genes. The light green and purple lines represent non-gene coding region of perennial ryegrass and *Epichloë* species. The grey line and pink breaking line show the location of the intron and aryl-phospho-beta-D-glucosidase domain, respectively. (**c**) Genetic linkage map of perennial ryegrass LG5 with the *Lp*BGNL-related locus. The *Lp*BGNL-related marker locus is indicated with the red arrow. Genetic distance (cM) is shown on the right side of the genetic markers.

An in-house bacterial artificial chromosome (BAC)-based genomic library of perennial ryegrass had previously been constructed from endophyte-devoid (E^−^) individuals of the cultivar Grasslands Nui. PCR-based screening of the library identified two positive clones, designated *Lp*BAC94-B20 and *Lp*BAC125-N24. *De novo* sequence analysis and assembly identified the presence of *Lp*BGNL in both clones. The gene was located within 39 kb- and 24 kb-contigs (NCBI UI: KY771171 and KY771172) of *Lp*BAC94-B20 and *Lp*BAC125-N24, respectively, along with a sequence (ca. 2 kb in length) showing similarity at a DNA sequence identity of 82% to a *Zea mays* transposon-related gene (NCBI UI: AF434192.1) (Fig. 2a). A 11 kb-contig of *E*. *festucae* genome sequence (NCBI UI: EF015481), which includes the corresponding ß-1,6-glucanase gene, was obtained from the NCBI database and was shown to contain three other genes located within a 5 kb distance from the ß-1,6-glucanase gene. In the BAC clone-derived contigs, however, no sequences similar to these flanking gene were identified. Putative coding regions for the *E*. *festucae* ß-1,6-glucanase and *Lp*BGNL genes were identified (Fig. 2b). A single intron was found in both sequences, and comparison of the exonic and intronic regions identified 4 insertion-deletion (indel) variations between them. Although the position of the intron was conserved, it seemed that almost all intron sequence was replaced in perennial ryegrass, due to insertion and deletion events. Although the coding regions were relatively highly conserved, no sequence similarity was found in the flanking sequence of the coding regions. A BLAST search of 1.5-kb upstream and downstream sequences of *Lp*BGNL identified partial sequence similarity to the genomes of wheat and rice, while the corresponding upstream and downstream sequences of the *E*. *festucae* ß-1,6-glucanase gene included partial sequences of the flanking genes (Fig. 2b).

Sequencing of PCR products generated using *Lp*BGNL-specific primers identified a 51-bp intron-located polymorphism between haplotypes of the heterozygous parent (C3 genotype) of the perennial ryegrass p150/112 genetic linkage mapping population (Supplementary Fig. S1), which facilitated development of an indel-based DNA marker. From the p150/112 population, 48 individuals were genotyped (Supplementary Fig. S2), and the *Lp*BGNL-related marker locus was assigned to a distal region of perennial ryegrass linkage group (LG) 5 (Fig. 2c).

### LpBGNL gene expression analysis

**Figure 3:**
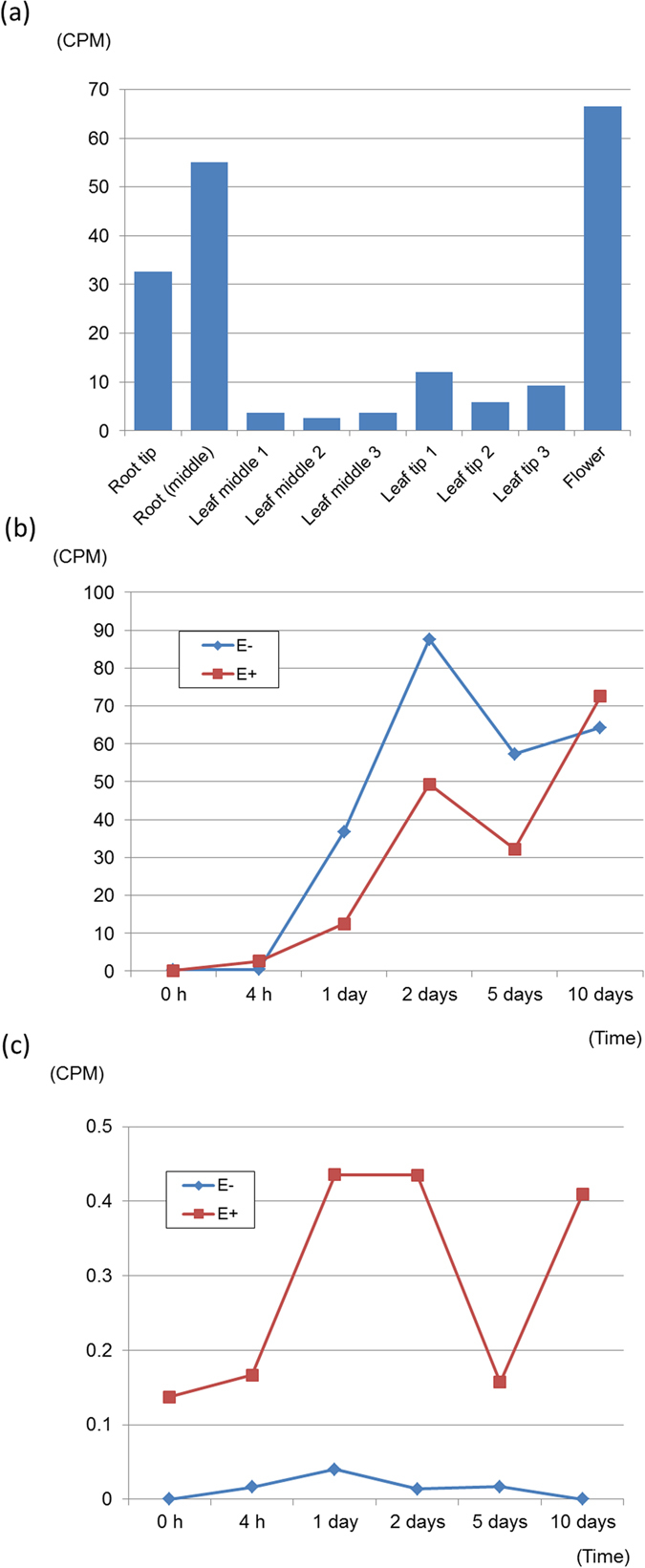
The expression levels of *Lp*BGNL and the *E*. *festucae* var. *lolii* ß-1,6-glucanase gene. (**a**) The expression level of *Lp*BGNL in each tissue of perennial ryegrass. The y-axis shows normalised read count number (CPM). (**b** and **c**) The expression levels of the genes in E^−^ and E^+^ perennial ryegrass genotypes. The x-axis shows time since the germination treatment, and y-axis shows CPM for *Lp*BGNL (**b**) and the *E*. *festucae* var. *lolii* ß-1,6-glucanase gene (**c**).

Expression of *Lp*BGNL was determined using data from the transcriptome sequence of the Impact_04_ genotype (NCBI BioProject Accession: PRJNA379202). Sequencing reads corresponding to *Lp*BGNL were identified from leaf, root and flower samples, and higher expression levels [based on counts per million reads (CPM)] were detected in root and flower than in leaf (Fig. 3a and Supplementary Table S2). Specificity of gene expression was examined using endophyte-infected (E^+^) and E^−^ perennial ryegrass seeds and seedlings. Due to sequence divergence, sequencing reads corresponding to the plant and fungal gene could be reliably discriminated (Supplementary Table S3). Read counts were very low for both E^+^ and E^−^ seeds immediately after the germination treatment (Fig. 3b). Although no large-scale morphological change was observed during the following two days, the read counts substantially increased in both samples. The counts remained at relatively high levels in young seedlings at 5 and 10 days after treatment. As similar trends were observed for both E^+^ and E^−^ genotypes, presence of endophyte did not significantly affect *Lp*BGNL expression pattern. Expression of the endogenous *E*. *festucae* var. *lolii* ß-1,6-glucanase gene was observed in E^+^ seedlings, but the read count approach revealed relatively low levels throughout the 10 days, in contrast to *Lp*BGNL (Fig. 3c and Supplementary Table S3).

### Phylogenetic analysis of plant and fungal ß-1,6-glucanase(-like) genes

**Figure 4:**
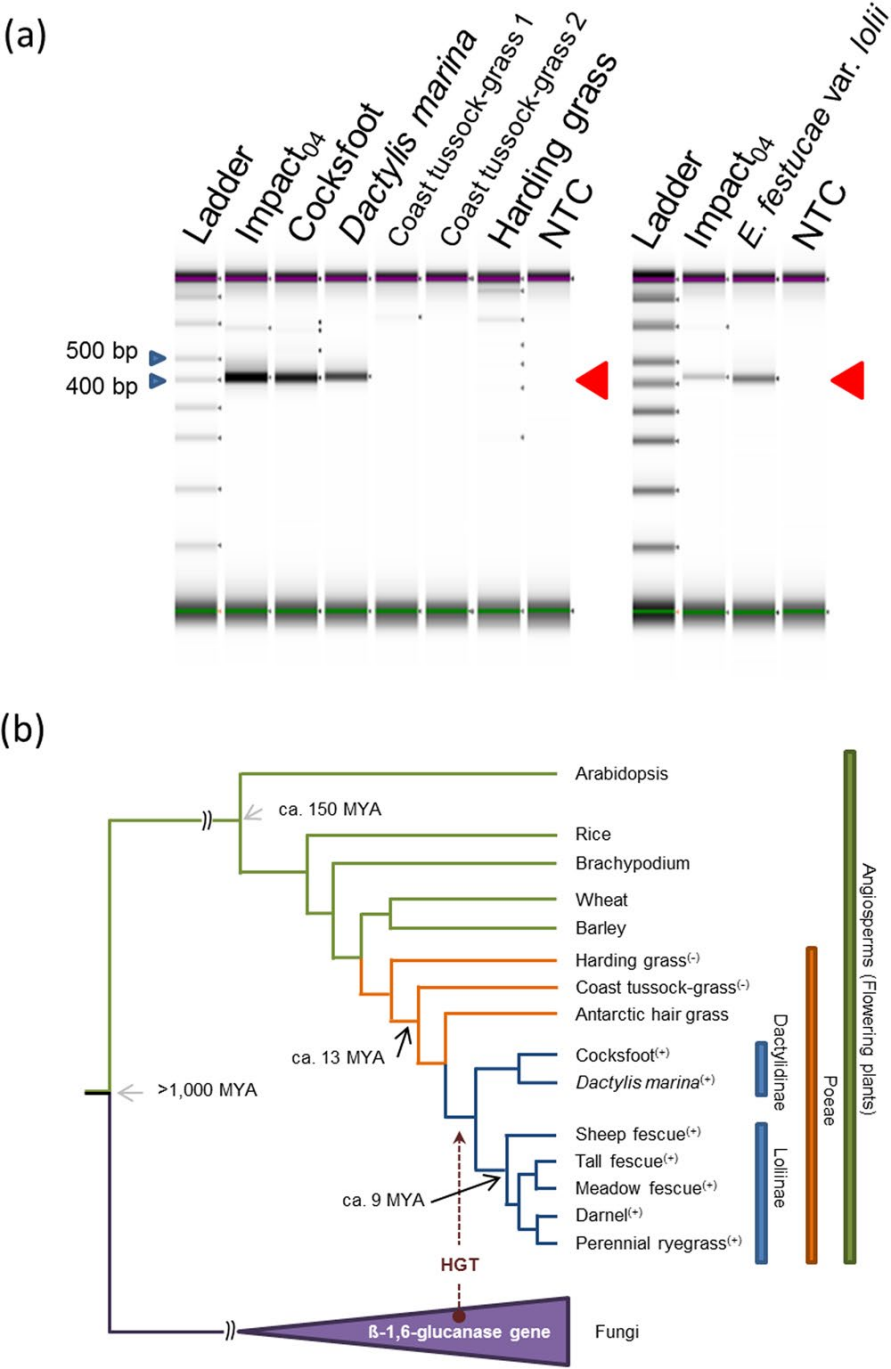
Phylogenetic tree of *Lp*BGNL orthologues and fungal ß-1,6-glucanase genes. The phylogram was generated based on amino acid sequence of the aryl-phospho-beta-D-glucosidase domain, and sequences from angiosperm and fungi species are indicated with green and purple, respectively. Asterisk (*) denotes species from which gene products have been confirmed to have the ß-1,6-glucanase activity. For cocksfoot, three contigs (haplotypes 1–3) were generated, and amino acid sequences from those contigs were used. For *Dactylis marina*, the contig without a putative premature stop codon was used. Strain and sequence contig (scaffold) identifiers of the Genome Project at the University of Kentucky website are shown in brackets. For the other fungi sequences, NCBI UI is shown in bracket. The clade including the sequences from *Trichoderma* and *Hypocreaas* species were selected as an outer group to obtain a root of the phylogenic tree. A figure legend generated with the MEGA7 software can be found in Supplementary Note S1.

The presence of *Lp*BGNL orthologues in other Poeae species was determined by PCR-based screening. *Lp*BGNL-specific primers were designed and short DNA fragments (178 bp in length) were amplified from genomic DNA templates of darnel (*Lolium temulentum* L.), meadow fescue (*Festuca pratensis* Huds.), tall fescue (*Festuca arundinacea* Schreb.), sheep fescue (*Festuca ovina*) (Table 1 and Supplementary Fig. S3). Products were also obtained from those of cocksfoot/orchard grass (*Dactylis glomerata* L.) and *Dactylis marina* Borrill, but not from those of coast tussock-grass [*Poa poiformis* (Labill.) Druce] or harding grass/phalaris (*Phalaris aquatica* L.). DNA fragments were not amplified from genomic DNA templates of *E*. *festucae var*. *lolii*., confirming the specificity of the oligonucleotide primers that were used. Alignment of *Lp*BGNL and the fungal genes identified a conserved region ca. 750 bp in length, corresponding to the aryl-phospho-beta-D-glucosidase domain. For a phylogenetic analysis, DNA fragments, including the aryl-phospho-beta-D-glucosidase domain, were amplified from the selected Loliinae and Dactylidinae species. *De novo* amplicon sequence analysis and assembly obtained a single sequence contig of the 750 bp region for each *Lolium* and *Festuca* species (Supplementary Fig. S4). For each *Dactylis* species, three contigs (haplotypes) were generated. A putative premature stop codon was found in two haplotypes of *Dactylis marina*, and the haplotypes with the premature stop codon were excluded from the further analysis. Fungal ß-1,6-glucanase gene-like sequences were obtained from the NCBI database and the Genome Project at the University of Kentucky website. Phylogenetic analysis with the maximum likelihood method was performed, and the plant species-derived sequences were found to be clustered in the phylogram with those from *Epichloë* (*Neotyphodium*) species (Fig. 4 and Supplementary Note S1). The sequences from other fungi were more distantly related to the plant sequences.

**Table 1:** Plant materials used in the current study.

Species		Genotype or cultivar	UI	Reference
Common name	Scientific name			
Perennial ryegrass	*Lolium perenne* L.	Impact_04_		Shinozuka *et al*., 2017
Darnel	*Lolium temulentum* L.	Genotype from Aberystwyth (Great Britain)	IBERS: BA13157	Hand *et al*.^37^
Meadow fescue	*Festuca pratensis* Huds.	Genotype from Tadham Moor (Great Britain)	IBERS: BF1199	Hand *et al*.^37^
Tall fescue	*Festuca arundinacea* Schreb.	Demeter		
Tall fescue	*Festuca arundinacea* Schreb.	Quantum		Hand *et al*.^37^
Sheep fescue	*Festuca ovina*	Genotype from Ponterwyd (Great Britain)	IBERS: BL2643	Hand *et al*.^37^
Cocksfoot (Orchard grass)	*Dactylis glomerata* L.	Currie	SARDI: 778	
(*Dactylis marina*)	*Dactylis marina* Borrill	Wild genotype from Algeria	SARDI: 38013	
Coast tussock-grass	*Poa poiformis* (Labill.) Druce	Wild genotype from Australia	SARDI: 41525	
Harding grass (Phalaris)	*Phalaris aquatica* L.	Landmaster		

UI denotes the unique identifier of the Genetic Resources Unit of Institute for Biological, Environmental and Rural Studies (IBERS; Aberystwyth, Wales. UK) or the South Australian Research and Development Institute (SARDI).

Synonymous and non-synonymous nucleotide substitution (K_s_/K_a_) ratios were calculated using the 750 bp sequences. Both K_s_ and K_a_ values between the *Epichloë* and plant species were substantially lower than those between the *Epichloë* species and other fungi (*H*. *lixii* and *T*. *harzianum*) (Table 2). The K_s_/K_a_ ratios for candidate *Lp*BGNL orthologues were between 0.027–0.221, lower or equivalent to values from the fungal ß-1,6-glucanase genes (0.057–0.275).

**Table 2:** Ks and Ka values, and Ka/Ks ratio within the Aryl-phospho-beta-D-glucosidase domain region between ß-1,6-glucanase(-like) gene sequences.

Ks, Ka (Ka/Ks)	Candidate orthologue of *Lp*BGNL				ß-1,6-glucanase gene			
	Darnel ryegrass	Tall fescue	Meadow Fescue	Sheep fescue	*E*. *festucae*	*Neotyphodium sp*.	*H*. *lixii*	*T*. *harzianum*
Perennial ryegrass	0.0622, 0.0017 (0.0273)	0.1302, 0.0102 (0.0783)	0.0991, 0.0102 (0.1029)	0.3333, 0.0416 (0.1247)	0.1961, 0.0501 (0.2553)	0.2539, 0.0629 (0.2477)	0.6116, 0.1507 (0.2464)	0.6396, 0.1469 (0.2297)
Darnel ryegrass	—	0.1670, 0.0085 (0.0509)	0.1359, 0.0085 (0.0625)	0.3575, 0.0433 (0.1211)	0.1957, 0.0518 (0.2647)	0.2533, 0.0646 (0.2550)	0.6041, 0.1491 (0.2468)	0.6383, 0.1453 (0.2276)
Tall fescue	—	—	0.0308, 0.0068 (0.2213)	0.3313, 0.0467 (0.1411)	0.1887, 0.0535 (0.2838)	0.2369, 0.0655 (0.2766)	0.6285, 0.1470 (0.2339)	0.6563, 0.1432 (0.2182)
Meadow Fescue	—	—	—	0.3247, 0.045 (0.1387)	0.1823, 0.0519 (0.2845)	0.2305, 0.0638 (0.2770)	0.6093, 0.1487 (0.2441)	0.6494, 0.1449 (0.2231)
Sheep Fescue	—	—	—	—	0.2207, 0.0433 (0.1961)	0.2969, 0.051 (0.1717)	0.6598, 0.1442 (0.2186)	0.6805, 0.1407 (0.2068)
*Epichloe festucae*	—	—	—	—	—	0.0896, 0.0247 (0.2751)	0.6363, 0.1488 (0.2339)	0.6424, 0.1492 (0.2323)
*Neotyphodium sp*.	—	—	—	—	—	—	0.6546, 0.1533 (0.2341)	0.6670, 0.1537 (0.2304)
*Hypocrea lixii*	—	—	—	—	—	—	—	0.1186, 0.0068 (0.0572)

**Figure 5:**
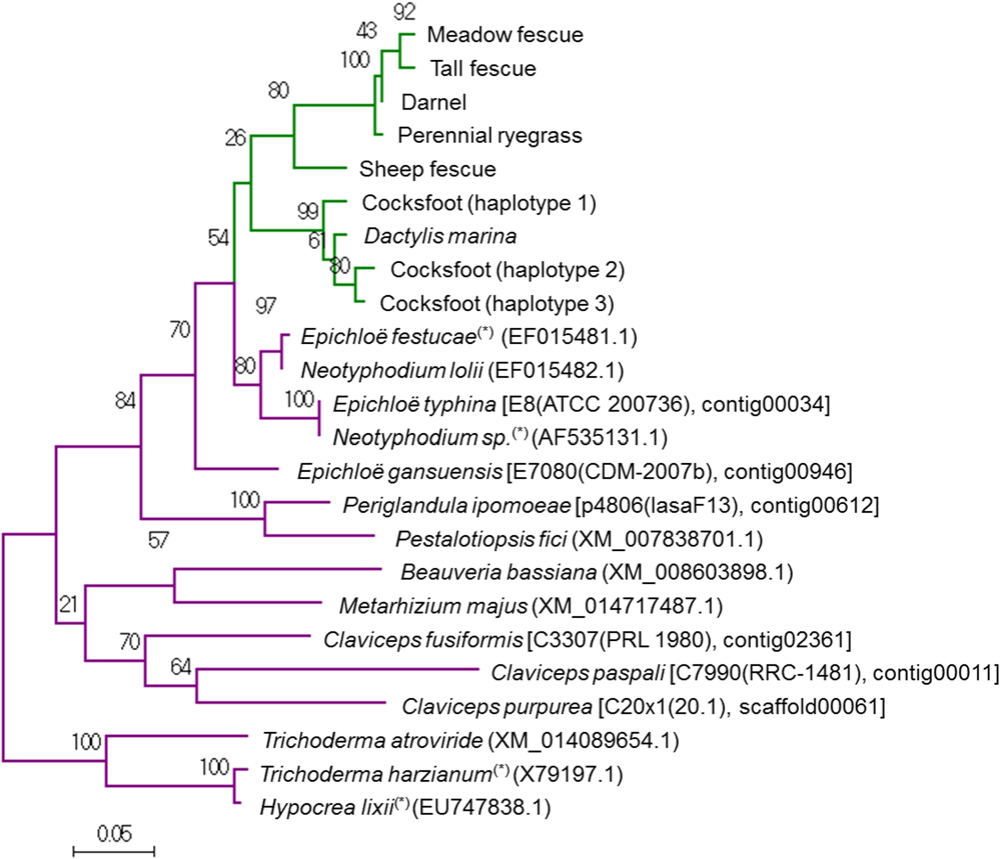
PCR screening for the *Lp*BGNL sequence, and taxonomic classification of plant species described in the current study (**a**) PCR amplicons from perennial ryegrass genotype Impact_04_, cooksfoot, *Dactylis marina*, coast tussock-grass and harding grass, and a strain of *E*. *festucae* are visualised on the 2200 TapeStation instrument (Agilent Technologies, CA, USA). The target fragments are indicated with the red arrow. Two genotypes of coast tussock-grass were subjected to the screening. The purple and green lines show the position of upper and lower markers, respectively, of the D1000 Kit (Agilent). NTC denotes no-template control for the PCR assay. The blue arrows indicate the positions of 400 and 500-bp fragments of the ladder. (**b**) Species classified into angiosperms, the tribe Poeae, and sub-tribes Loliinae and Dactylidinae are indicated by the green, orange and blue boxes, respectively, on the right side of the phylogenetic tree. The species which were positive and negative in the PCR screening step are indicated by the plus (+) and minus (−) signs, respectively. The divergent points of plant species from other species (>1,000 MYA) and the clade monocotyledon from other plant species (ca. 150 MYA) are indicated with light grey arrows. The divergent points of sub-tribes Loliinae and Dactylidinae from the remaining Poeae species, especially from genus *Poa*, which is a closely related taxon (ca. 13 MYA), and fine-leaved fescue (Sheep fescue) from broad-leaved fescues (tall fescue and meadow fescue) (ca. 9 MYA) are indicated with black arrows. The purple arrow represents the putative period of HGT in the evolutionary lineage.

In order to verify presence/absence boundaries, a set of PCR primers was designed to amplify 415 bp fragments within a region highly conserved between plant and fungal ß-1,6-glucanase(-like) genes. This assay confirmed the absence of the gene in the *Poa* and *Phalaris* species samples (Fig. 5). As a control experiment for capacity to amplify cross-species, PCR was performed with primers specific to perennial ryegrass histone H3 and candidate plant architecture genes, such as the ATP-binding cassette protein sub-family G 5 and 6 genes (*Lp*ABCG5 and *Lp*ABCG6, respectively). Although all primers were designed based on perennial ryegrass sequence, PCR amplification from coast tussock-grass and harding grass/phalaris was observed, except for the combination of ABCG 5 primers and one of the *Poa* genotypes (Supplementary Fig. S5). Database searches were performed using published short-read sequencing data on the NCBI Sequence Read Archive (SRA; https://www.ncbi.nlm.nih.gov/sra). Sequences significantly matching the *Lp*BGNL and *E*. *festucae* ß-1,6-glucanase genes were found from *Dactylis* species, but not from *Poa* or *Phalaris* species (Table 3, Supplementary Table S4). No significantly matching sequence was obtained from Antarctic hair grass (*Deschampsia antarctica* É. Desv.), which is believed to be taxonomically closer than *Poa* species to members of the sub-tribes Loliinae and Dactylidinae^1^ (Fig. 5b). As a control analysis, sequences similar to the *Lp*ABCG 5 and 6 genes were sought, leading to identification of matching sequences from all tested Poeae species, including those belonging to both *Poa* and *Phalaris*.

**Table 3:** Database search result using NCBI BLASTN suite. The sequence identity (%) and expect (E) value of the top matching sequence are shown.

Species	UI	Source	Data size	*Lp*BGNL	*E*. *festucae* ß-1,6-glucanase gene	*Lp*ABCG5	*Lp*ABCG6
*Dactylis glomerata* L. (orchardgrass)	SRX738187	Transcriptome	32.6G bases	95% (8e-35)	99% (6e-41)	99% (3e-41)	94% (5e-32)
*Deschampsia antarctica*	SRX465632	Genome	31G bases	N.S.	N.S.	99% (7e-42)	97% (1e-38)
*Poa annua*	SRX745831	Transcriptome	25.9G bases	N.S.	N.S.	100% (5e-43)	95% (1e-33)
*Poa supina*	SRX745855	Transcriptome	8.9G bases	N.S.	N.S.	99% (8e-42)	90% (6e-27)
*Poa infirma*	SRX745858	Transcriptome	9.3G bases	N.S.	N.S.	100% (2e-43)	92% (2e-26)
*Phalaris aquatica*	SRX669405	Transcriptome	10.2G bases	N.S.	N.S.	97% (5e-39)	94% (4e-34)

## Discussion

The high level of DNA sequence similarity to the fungal genes, and absence of *Lp*BGNL-like sequences from other representative angiosperm species suggested that *Lp*BGNL was obtained from a fungal species through HGT. However, it was also possible that the *Lp*BGNL sequence may have originated from a microbe associated with the plant individual, especially from a species of asexual fungal endophyte, such as *E*. *festucae* var. *lolii* (syn. *Neotyphodium lolii*), which are known to form symbiotic associations with perennial ryegrass^3^. Although an E^−^ plant individual was selected for sequencing on the basis of negative PCR-based screening for diagnostic DNA sequences, a fungal symbiont could be present in plant material below the limit of detection. Genomic and genetic characterisation was consequently performed in order to demonstrate that *Lp*BGNL is located on a perennial ryegrass chromosome. Furthermore, *Lp*BGNL orthologues were found to be present in other Loliinae and Dactylidinae species. It is hence unlikely that *Lp*BGNL was an assembly or annotation artefact, even though *Lp*BGNL shows unusually high DNA sequence similarity (ca. 90%) to the ß-1,6-glucanase genes of contemporary species descended from the putative donor, when compared to other horizontally transferred genes in eukaryotes^20^.

The PCR-based screening and database searches suggested that the ß-1,6-glucanase-like gene is present in only a limited number of Poeae species including the genera *Lolium*, *Festuca* and *Dactylis*, which are confined to the sub-tribes Loliinae and Dactylidinae^1, 21^. The phylogenetic analysis suggested a common origin of the *Epichloë*-derived ß-1,6-glucanase genes and *Lp*BGNL orthologues, and the close relationship between the *Lp*BGNL orthologues of contemporary Loliinae and Dactylidinae grasses suggests that the gene may have been introduced into the genome of a common ancestor of the sub-tribes by a single transfer event. The HGT event may consequently have occurred between ca. 9 to 13 million years ago (MYA), based on the predicted time of divergence of the two sub-tribes from other Poeae lineages (Fig. 5b)^21–23^.

Despite the relatively recent appearance (ca. 200–230 MYA) and diversification (ca. 100–125 MYA) of the lineage, the angiosperms currently compose the largest grouping of land plants, with nearly 300,000 species^24–26^. In the previous systematic study of four angiosperm genomes (*A*. *thaliana*, rice, sorghum and poplar) no evidence for HGT from fungal species was found^16^. Due to identification of two and three events for moss and lycophyte lineages, respectively, it was consequently concluded that gene transfer between fungi and angiosperms must be exceedingly infrequent. The present study, however, has demonstrated that such an event has also occurred relatively recently in evolutionary time. Considering the more distant date of divergence of the moss and lycophyte lineages (>400 MYA) compared to angiosperm species^24^, a longer period of evolutionary time may have provided more opportunities for fixation of transfer events in moss and lycophyte species. The specificity of the ß-1,6-glucanase-like gene to only two sub-tribes of a single family suggests the possibility that other fungal-angiosperm HGT events could also be highly lineage-specific, and their detection would require more detailed comparisons of a larger number of fully sequenced genomes.

The Ks/Ka ratios between plant ß-1,6-glucanase-like genes (0.027–0.221) were not substantially different from those of the fungal ß-1,6-glucanase genes, and those of *Lp*ABCG5 and *Lp*ABCG6 (0.166 and 0.238)^27^. This suggests that *Lp*BGNL may have been subjected to selection pressures. Although similar Ks/Ka ratios were obtained from the angiosperm and fungi groups, DNA mutation rates between those two groups may not be equivalent. A further characterisation is essential to confirm that both gene groups have been subjected to selection pressure at an equivalent level. From cocksfoot/orchard grass and *Dactylis marina*, three haplotypes of the aryl-phospho-beta-D-glucosidase domain were identified, and two haplotypes obtained from *Dactylis marina* contained a putative premature stop codon. As these *Dactylis* species have autopolyploid genomes^28^, the ß-1,6-glucanase-like genes in these species may have been subjected to unique selection pressures due to genetic redundancy, when compared with genes in the other plant species.

As asexual symbiotic *Epichloë* species grow as hyphae between cells of vegetative aerial tissues in Poeae species^3, 29^, it is likely that the close physical proximity of both partners in the symbiosis may have facilitated an HGT event, similar to the physical contacts between parasitic and host plants^7, 9^. The conservation of the intron position between the *Lp*BGNL and *Epichloë* ß-1,6-glucanase genes suggests that a part of the endophyte genome including the ß-1,6-glucanase gene, rather than a reverse-transcription product of endophyte gene mRNA, was incorporated into the recipient genome. In prokaryotes, transformation is a prevalent mechanism of gene exchange, in which HGT occurs through uptake of exogenous double-stranded DNA by the recipient cell^9^. It is possible that the ancestral *Lp*BGNL gene was acquired through a similar mechanism, due to endophyte cell death and transient liberation of genomic DNA in the vicinity of recipient plant cells. This scenario suggests that a transformation-like mechanism may have been active in the angiosperm lineages, as well as other HGT mechanisms such as *Agrobacterium* spp.-based conjugation and endosymbiotic gene transfer^9^.

In multicellular organisms, foreign genes introduced during unicellular or early developmental stages may increase the chance of successful transmission to offspring, due to subsequent potential dispersion of the genes within the individual at maturity, including to germline cells (‘weak-link model’)^30^. The life-cycle of asexual *Epichloë* endophytes involves growth within plant reproductive tillers and colonisation of inflorescences (including intimate contact with female gametophytic structures), followed by maternal transmission through seed^3^ It is consequently possible that the ß-1,6-glucanase-like gene was introduced into an ancestral angiosperm species from infecting fungi during an early developmental stage, resulting in successful transmission into offspring. Symbiotic and parasitic relationships are thought to have been important factors for facilitation of HGT between eukaryote genomes^7, 9^. Considering the importance of event timing during the life-cycle of recipients, additional ancestral HGT events in angiosperm genomes may be detected if the species associates with symbiotic or parasitic partners during unicellular or early developmental stages, such as entomophilous flowers which interact with insect pollen vectors and microorganisms commensal with insects^31^.

Fungal ß-1,6-glucanases have been reported to be specifically secreted into plant apoplasts during endophyte infection, and may play a role in provision of nutrition to the infecting endophyte, control of branching of the endophyte hyphae, and protection of plant tissues from infection of other fungal pathogens^29^. It is possible that the plant-encoded enzyme may participate in one or more of these processes, and so contribute to establishment of a stable symbiotic relationship. Although *Epichloë* endophytes do not colonise root tissues^32^, a relatively high level of expression of *Lp*BGNL was observed in root tissues. It is hence possible that the plant-encoded enzyme may function to protect against infection by soil-borne fungal pathogens. Similarly, active expression in flowers may suggest the capacity to protect against fungal pathogens such as *Epichloë typhina*, which causes choke disease^33^. Further analysis is, however, required to test this hypothesis, as some *Festuca* and *Dactylis* species are relatively susceptible to infection by *E*. *typhina*, even though those species presumably also possess the ß-1,6-glucanase-like gene^4, 33, 34^. As natural stable associations with asexual *Epichloë* endophytes are confined to the Poeae lineages that possess *Lp*BGNL-like genes, the ancestral HGT event (which might have occurred from a sexual pathogenic *Epichloë*-like species) may have provided pre-adaptive conditions for the contemporary symbiosis. The evidence for selective pressure on the gene is suggestive, but further functional analysis is required to fully test this hypothesis.

As major symbionts, the asexual *Epichloë* species provide abiotic and biotic stress tolerance to grass species of the Poeae tribe. Tolerance to invertebrate herbivory is a well-characterised benefit to the host, partially attributable to the effects of a *makes caterpillars floppy-like* (*mcf-like*) gene^6^. The *mcf*-like gene was horizontally transferred into the endophyte genome from a bacterial species 7.2–58.8 MYA. It is hence possible that multiple horizontal transfer events, including transfer of the *Lp*BGNL-like gene as described in the present study, have been involved in the establishment of the current stable symbiotic relationship.

The potential role of a ß-1,6-glucanase-like gene in protection against other, pathogenic, fungal species is of particular interest. Species such as *T*. *harzianum* are mycoparasites of fungal phytopathogens, and this property is related to glucanase activity^35^. Fungal-derived genes for anti-fungal enzymes such as endochitinases and glucanases have also been used for generation of transgenic plants with enhanced pathogen resistance^35, 36^. Further studies of the role of the *Lp*BGNL-like gene could hence involve experimental transfer into the genomes of crop plants such as rice and wheat, and subsequent evaluation of resistance to fungal diseases.

## Methods

### Plant materials and DNA extraction

Details of plant genotypes are summarised in Table 1 ^19, 37^. Genomic DNA was extracted from young leaves of plants and fungal endophyte mycelium using the DNeasy plant mini kit (QIAGEN, Hilden, Germany).

### PCR amplification

Locus-specific primers were designed using the Sequencher software (GENECODE, MI, USA) and the PCR primers are listed in Supplementary Table S5 ^27, 38^. PCR amplification was performed with MyFi polymerase kit (BIOLINE, London, UK). PCR amplicons were visualised on the 2200 TapeStation instrument.

### Short-read sequencing of BAC clones and amplicons

The BAC-based genomic library^39^ was screened through use of PCR. For the phylogenomic analysis, PCR primers were designed to obtain genomic fragments from Loliinae and Dactylidinae species (Supplementary Table S5). Sequencing libraries for the MiSeq platform (Illumina, San Diego, California, USA) were prepared from the BAC clones and PCR amplicons, following the previously described MspJI-based method^40^. The library was characterised with the TapeStation and Qubit instruments (Thermo Fisher Scientific). The outcome reads were assembled with the Sequencher and SOAPdenovo programs^41^.

### Genetic linkage analysis

PCR primers were designed to detect the indel polymorphism within the *Lp*BGNL sequence (Supplementary Table S4). Genetic linkage analysis was performed through use of the p150/112 reference genetic mapping population of perennial ryegrass using the JoinMAP 3.0 application^42, 43^.

### Gene expression analysis

The transcriptome sequencing reads from Impact_04_ tissues were mapped against Impact_04_ genome contigs (>999 bp) for filtering. The number of reads which contained *Lp*BGNL sequence (no sequence mismatch for 60 bp or longer) were counted as *Lp*BGNL-derived reads. For gene expression in seedlings, E^+^ and E^−^ seeds of perennial ryegrass cultivar Alto were subjected to germination treatment by placement on wet filter paper in the dark for 2 days followed by seedling growth under full-light conditions^44^. RNA was extracted with a CTAB extraction method, and sequencing libraries were prepared using the SureSelect strand-specific RNA library preparation kit (Agilent). Sequencing analysis was performed on the Illumina HiSeq 3000 platform.

### *In silico* analysis

The DNA sequences of fungal ß-1,6-glucanase genes were obtained from the NCBI (http://www.ncbi.nlm.nih.gov/) database and the Genome Project at the University of Kentucky website^45^. Putative orthologous sequences were sought in the NCBI, *Brachypodium distachyon* (http://www.brachypodium.org/) and Ensembl (http://plants.ensembl.org/index.html) databases. Non-synonymous and synonymous nucleotide substitution rates (Ka and Ks, respectively) were calculated using the Synonymous Non-synonymous Analysis Program (SNAP; http://www.hiv.lanl.gov/). Alignment of DNA sequences was performed with the CLUSTALW program (http://www.genome.jp/tools/clustalw/) with the default parameters. Phylogeny was generated with the MEGA7 program (http://www.megasoftware.net/).

### Data Availability

The datasets of perennial ryegrass transcriptome sequencing are available in the NCBI SRA repository, (https://www.ncbi.nlm.nih.gov/bioproject/PRJNA379202). The other sequencing datasets generated during and/or analysed during the current study are available from the corresponding author on reasonable request.

## Electronic supplementary material

Supplementary Information
