# Supplementary material for: Horizontal transfer of a ß-1,6-glucanase gene from an ancestral species of fungal endophyte to a cool-season grass host
Source: Sci Rep. 2017 Aug 22;7:9024. doi: 10.1038/s41598-017-07886-2 (PMC5567365; doi:10.1038/s41598-017-07886-2)
Supplement: Supplementary file 1 — Supplementary Information [file 41598_2017_7886_MOESM1_ESM.pdf]

Horizontal transfer of a ß-1,6-glucanase gene from an ancestral species of fungal endophyte to a cool-season grass host

Hiroshi Shinozuka[1,2,*], Inoka K Hettiarachchige[1,2], Maiko Shinozuka[1,2], Noel O.I. Cogan[1,2,3], German C. Spangenberg[1,2,3], Benjamin G. Cocks[1,2,3] John W. Forster[1,2,3] Timothy I. Sawbridge[1,2,3]

[1]Agriculture Victoria, AgriBio, Centre for AgriBioscience, 5 Ring Road, La Trobe University, Bundoora, Victoria 3083, Australia
[2]Dairy Futures Cooperative Research Centre, Australia
[3]School of Applied Systems Biology, La Trobe University, Bundoora, Victoria 3086, Australia
[*]Corresponding author: e-mail – hiroshi.shinozuka@ecodev.vic.gov.au

Supplementary Information Legends

Supplementary Fig. S1
DNA sequence alignment of the two haplotypes from the C3 genotype. Dash (-) in the DNA sequence shows a gap, and asterisk (*) under the alignment denotes ‘conserved nucleotide’. DNA sequence corresponding to the PCR primers for the indel-based genotyping assay is underlined.

Supplementary Fig. S2
Result of the indel-based genotyping assay. The PCR amplicons were visualised on an agarose gel including the SYBR™ Safe DNA Gel stain (Thermo Fisher Scientific, Waltham, Massachusetts, USA). EasyLadder I (BIOLINE) was used as a size standard, and the 250 and 500 bp fragments are indicated with red arrows. The genotype UI of the genetic mapping population is indicated in each lane. C3 and NTC stand for ‘the C3 genotype’ and ‘no-template control’.

Supplementary Fig. S3
PCR screening for the *Lp*BGNL sequence using the *Lp*BGNL locus-specific primers (LpBGNL_short). (a) PCR amplicons from perennial ryegrass genotypes $Impact_{04}$ and C3, and perennial ryegrass-associated endophyte were visualised on the 2200 TapeStation instrument using the High Sensitivity D1000 Kit (Aglient). The purple and green lines show the position of upper and lower markers, respectively, of the High Sensitivity D1000 Kit. (b) PCR amplicons from *Lolium* and *Festuca* species (sub-tribe Loliinae) were visualised on an agarose gel containing the SYBR™ Safe stain. Tall fescue genotypes from two cultivars (Demeter and Quantum) were subjected to the screening. NTC denotes ‘no-template control’ for the PCR assay. (c) PCR amplicons from cocksfoot, *Dactylis marina*, coast tussock-grass and harding grass were visualised on the 2200 TapeStation instrument using the D1000 Kit. The purple and green lines show the position of upper and lower markers, respectively, of the D1000 Kit. Two genotypes of coast tussock-grass were subjected, and the $Impact_{04}$ genotype was used as a positive control.

Supplementary Fig. S4
DNA sequences of the conserved ca. 750-bp region from plant species. Putative premature stop codons were found in *Dactylis marina* haplotypes 2 and 3.

Supplementary Fig. S5
PCR assay for confirmation of a cross-species amplification capacity. PCR amplicons from cooksfoot, *Dactylis marina*, coast tussock-grass and harding grass were visualised on the 2200 TapeStation instrument using the D1000 Kit. The purple and green lines show the position of upper and lower markers, respectively, of the D1000 Kit. The position of the target amplicons is indicated with a red arrow. The $Impact_{04}$ genotype was used as a positive control, and NTC denotes ‘no-template control’ for the PCR assay.

Supplementary Information Legends (Cont'd)

Supplementary Table S1
DNA sequence identity between *Lp*BGNL and fungi ß-1,6-glucanase genes. 'NCBI UI' denotes the unique identifier of the NCBI database.

Supplementary Table S2
Result of the read count-based gene expression analysis from tissue-specific sequencing libraries. The number of reads obtained through filtering is shown as 'total reads', and 'counts' denote the number of reads corresponding to *Lp*BGNL.

Supplementary Table S3
Result of the read count-based gene expression analysis from E- and E+ seedling-derived sequencing libraries. The number of reads obtained through filtering is shown as 'total reads', and 'counts' refer to the number of reads corresponding to the *Lp*BGNL or *Epichloë* ß-1,6-glucanase gene.

Supplementary Table S4
Details of the short-read sequencing dataset used in the current study.

Supplementary Table S5
PCR primers used in the current study.

Supplementary Note S1
Figure legend for Fig. 4 generated with the MEGA7 program.

Supplementary Fig. S1

```
C3-hap1      AGGGCATCAACAAGATCAGGGGCGTTAATTTCGGCGGTATGTCTTCTTTACAGCTGCTAC
C3-hap2      AGGGCATCAACAAGATCAGGGGCGTTAATTTCGGCGGTATGTCTTCTTTACAGCTGCTAC
             ************************************************************

C3-hap1      TTCCTCCATCCTCCGATAATACAAGTCACATTCTTTTTGCAAAATTATATGGATCCAGAA
C3-hap2      TTCCTCCGTCGTCCCATAATATAAGTCACATTCTTTTTCCAAAATTATATGTATCCAGAA
             ******* ** *** ****** **************** ************ ********

C3-hap1      -GTATAAATGGTTGCATTCC-TCTTACAGATCTCTTTCATTTCATATATGTTCATTCAGA
C3-hap2      CGTATAAATGTTTGCATTCTGTCTTGCAAATCTCTTTCATTTCATATATATTTATACAGA
              ********* ********  **** ** ******************** ** ** ****

C3-hap1      TTTTGTGGA----------------------------GTACT------------------
C3-hap2      TTTCGTGGATATATATTATAAGTGTAACTCATGGTAAATACTGTAAATTGTTGACCAGCA
             *** *****                             ****

C3-hap1      -----AACTAACAGAGGTTGAAATTCTCAGGCTGGCTCATCTGTGAGCCATGGATGATGA
C3-hap2      GCTAAATTTAACATGGGTTGAAATTCTCAGGCTGGCTCATCTGTGAGCCATGGATGATGA
                  *  *****  *********************************************

C3-hap1      GCGACG
C3-hap2      GCGACG
             ******
```

Supplementary Fig. S2

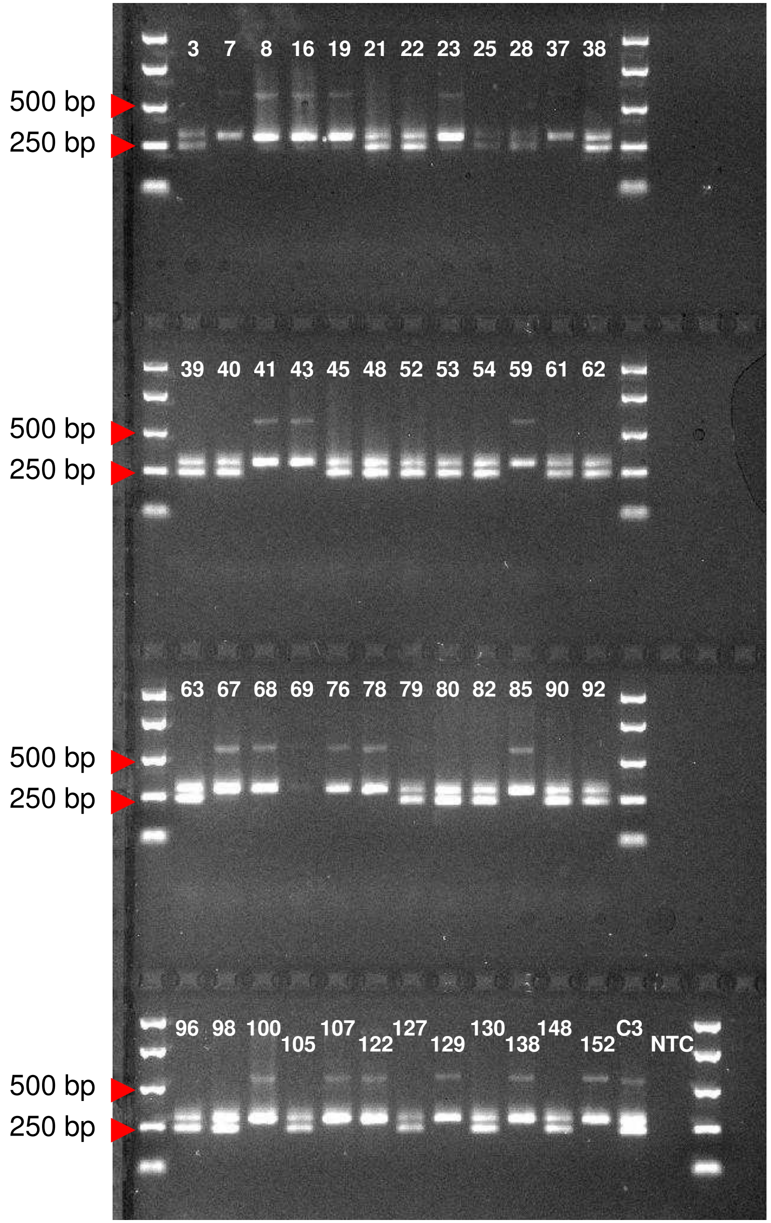

Supplementary Fig. S3

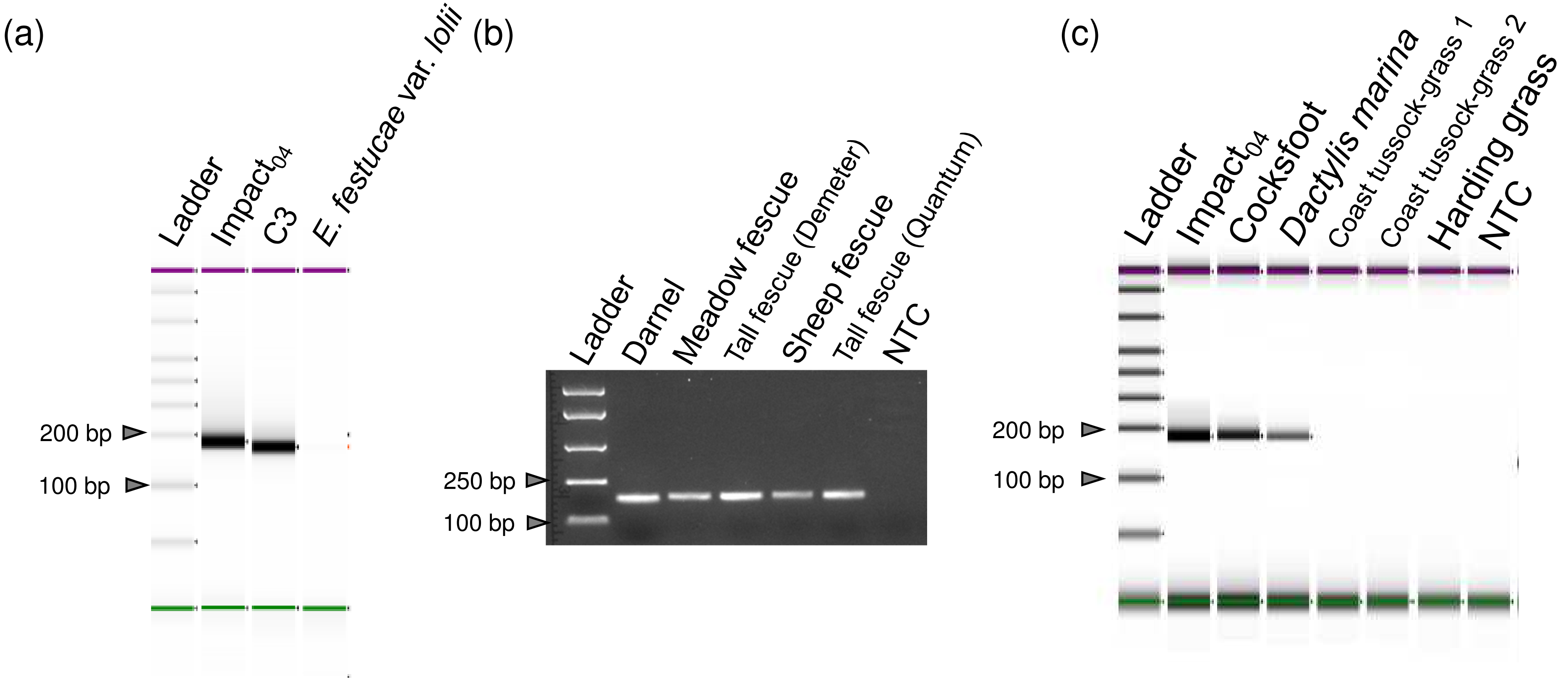

Supplementary Fig. S4

>Perennial ryegrass
ATGATGAGCGACGAGTGGAACAACGTCATGGGCTGCAACGGGGCTGCCTCCGAGTTCGACTGCATGCTAAACAATTACATGGGAAGCAATCGAGCTGCCGGCAACGACAAGTTCGAGACTCATTGGAGGACTT
GGATCAATGCCGACAGCGTCGAGTCAGTCCACTATGTTGGCCTGAACACGATTCGCATCCCCATCGGGTACTGGTCCTACGTGGACATTGTCGACAAGGCCAGTGAGCCCTTTGCCGATGGCAACAGGATGCT
CCCATACCTGGACGCCGTCGTCCAAAAGGCTGCTGACCTTGGCATGTATGTCATCATCGATCTTCATGGGGCTCCCGGCGGGCAGCAACAAGACGCCTTCACCGGCCAGAACAACAAGCCGGCCGGTTTCTTC
AACGACTACAACTTTGACCGCGCCAACAGGTGGATGTCGTGGATGACGAGGCGCATCCACACAAACCCTGCCTATGCCACCGTCGGCATGATTGAGGTTCTCAACGAGCCCGTGTCCGGACACGACTCGGAC
GGACGGTACCCTGCCCCCGGTCAGGTCCCCGGGCTGGTCCAGAAGTACTACCCGGGCGCTCTCAAGGCGGTCCGAGATGTCGAAGCGTCTCTCGGAGTGGCTGACGGCAACAAGCTCCATGTGCAGTTCAT
GTCCCAGAAGTGGGACTCGGGCAACGCGCGCGACAACTCTGCCGTGGCCAACGACCAACGCACTGCGTTCGATGACCACAACTACATT

>Meadow fescue
ATGATGAGCGACGAGTGGAACAACGTCATGGGCTGCAACGGGGCTGCCTCCGAGTTCGACTGCATGCTAAACAATTACATGGGAAGCAATCGAGCTGCCGGCAACGACAAGTTCGAGACTCACTGGAGGACT
TGGATCAATGCCGACAGCGTCGAGTCAGTCCACTATGTTGGCCTGAACACGATTCGCATCCCCATCGGGTACTGGTCCTACGTGGACATTGTCGACAAGGCCAGTGAGCCCTTTGCCGACGGCAACAGGATGC
TCGCATACCTGGATGCCGTTGTCCAAAAGGCTGCTGACCTTGGCATCTATGTCATCATCGATCTGCATGGGGCTCCCGGCGGGCAGCAACAAGACGCCTTTACCGGCCAGAACAACAAGCCGGCCGGTTTCTT
CAACGACTACAATTTTGACCGCGCCAACAGGTGGATATCGTGGATGACGAGGCGCATCCACACAAACCCTGCCTACGCCACCGTCGGCATGATTGAGGTTCTCAACGAACCCGTCTCCGGACACGACCCGGCC
GGACGGTACCCTGCCCCGGGTCAGGTCCCGGGGCTGGTCCAGAAGTACTACCCGGGCGCTCTCAAGGCGGTCCGAGATGCCGAAGCGTCTCTCGGCGTTGCTGACGGCAACAAGCTCCACGTGCAGTTCAT
GTCCCAGAAATGGGACTCGGGCAACGCGCGCGACAACTCTGCCGTGGCCAACGACCAACGCACTGCGTTCGATGACCACAACTACATT

>Tall fescue
ATGATGAGCGACGAGTGGAACAAGGTCATGGGCTGCAACGGGGCTGCCTCCGAGTTCGACTGCATGCTAAACAATTACATGGGAAGCAATCGAGCTGCCGGCAACGACAAGTTCGAGACTCACTGGAGGACT
TGGATCAATGCGGACAGCGTCGAGTCAGTCCACTATGTTGGCCTGAACACGATTCGCATCCCCATCGGGTACTGGTCCTACGTGGACATTGTGGACAAGGCCAGTGAGCCCTTTGCCGACGGCAACAGGATGC
TCCCATACCTGGATGCCGTTGTCCAAAAGGCCGCTGACCTTGGCATCTATGTCATCATCGATCTGCATGGGGCTCCCGGCGGGCAGCAACAAGACGCCTTTACCGGCCAGAACAACAAGCCGGCCGGTTTCTA
CAACGACTACAATTTTGACCGCGCCAACAGGTGGATATCGTGGATGACGAGGCGCATCCACACAAACCCTGCCTACGCCACCGTCGGCATGATTGAGGTTCTCAACGAACCCGTCTCCGGGCACGACCCGGAC
GGACGGTACCCTGCCCCGGGTCAGGTCCCGGGGCTGGTCCAGAAGTACTACCCGGGCGCTCTCAAGGCGGTCCGAGATGCCGAAGCGTCTCTCGGCGTTGCTGACGGCAACAAGCTGCACGTGCAGTTCAT
GTCCCAGAAATGGGACTCGGGCAACGCGCGCGACAACTCTGCCGTGGCCAACGACCAACGCACTGCGTTCGATGACCACAACTACATT

>Darnel
ATGATGAGCGACGAGTGGAACAACGTCATGGGCTGCAACGGAGCTGCCTCCGAGTTCGACTGCATGCTAAACAATTACATGGGAAGCAATCGAGCTGCCGGCAACGACAAGTTCGAGACTCATTGGAGGACTT
GGATCAATGCCGACAGCGTCGAGTCAGTCCACTATGTTGGCCTGAACACGATTCGCATCCCCATCGGGTACTGGTCCTACGTGGACATTGTCGACAAGGCCAGCGAGCCCTTCGCCGACGGCAACAGGATGC
TCCCATACCTGGACGCCGTCGTCCAAAAGGCTGCTGACCTCGGCATCTATGTCATCATCGATCTTCATGGGGCTCCCGGTGGGCAGCAACAGGACGCCTTTACCGGCCAGAACAACAAGCCGGCCGGTTTCTT
CAACGACTACAACTTTGACCGCGCCAACAGGTGGATGTCGTGGATGACGAGGCGCATCCACACAAACCCTGCCTATGCCACCGTCGGCATGATTGAGGTTCTCAACGAGCCCGTGTCCGGACACGACTCGGA
CGGACGGTACCCTGCCCCCGGTCAGGTCCCCGGGCTGGTCCAGAAGTACTACCCGGGCGCTCTCAAGGCGGTCCGAGATGTCGAAGCGTCTCTCGGAGTGGCTGACGGCAACAAGCTCCATGTGCAATTCA
TGTCCCAGAAGTGGGACTCGGGCAACGCGCGCGACAACTCTGCCGTGGCCAACGACCAACGCACTGCATTCGATGACCACAACTACATT

>Sheep fescue
ATGATGAGTGACGAGTGGAACAACGTCATGGGCTGCAACGGGGCTGCCTCCGAGTTCGACTGCATGCTGAACAATTACATGGGAACCAAACGAGCCCAAGGCAACGACAAATTCGAGACTCACTGGAGGACTT
GGATCAACGCCGACAGCGTCCAGTCAGCCCACGACGTCGGCCTCAACACGATACGCATTCCCATCGGGTACTGGTCCTACGTAGACATCGTCGACAAGGCCAGCGAGCCCTTTGCTGATGGCAACAGGATGC
TCCCCTACCTGGACGCCGTCGTCCAGAAGGCCGCCGACCTTGGCATGTACGTCATCATCGACCTGCACGGGGCACCCGGCGGCCAGCAGGAAGACCCCTTTACCGGCCAGAACAACAAGCCGGCCGGTTTC
TTCGACGACTACAACTTTGGCCGTGCCCAAAAGTGGATGTCGTGGATGACGAGGCGCATCCACACAAACCCTGCCTACGCCACCGTCGGCATGATTGAAGTACTGAACGAGCCCGTCTCCGGGCATGATGGG
GGCGGACGGTACCCTGCACCTGGTCAGGTCCCCGGGCTGGTCCAGAAGTACTACCCAGGCGCTCTGAAGGCGGTTCGAGACGCTGAAGCGTCGCTCGGCGTGGCTGACAGCAACAAGCTCCACGTGCAATT
CATGTCCCAAAAATGGGACTCCGGCAACCCACGCGACAGCTCAGCCGTGGCCAACGACAAGTTCACTGCGTTCGATGACCACAACTACATT

Supplementary Fig. S4 (Cont’d)

>Cocksfoot (haplotype 1)
ATGATGAGTGACGAGTGGAACAACGTCATGGGTTGCAGCGGGGCTGCCTCCGAGTTCGACTGCATGCTCAACAATTACGGCGGAAGTAAACGAGACGCCGGCAACGAGAAGTTCGAGACTCACTGGAGGACT
TGGATCAACGCCGACAGCGTCCAGTCAGCCCACGACGTTGGCCTGAACACCATTCGCATTCCCATGGGGTACTGGTCCAACGTAGACATCGTCGACAAGGCCAGCGAGCCCTTCGCCGACGGCGACAAGATG
CTCCCCTACCTGGACGCCGTCGTCCAGAAGGCCGCTGACCTGGGCATGTATGTCATCATCGATCTGCACGGGGCACCCGGAGGCCAGCAGGAGGACAACTTCACCGGCCAGAATAACAAGCCGGCCGGCTT
CTTCAACGACTACAACTTCGGCCGTGCCCAGGCATGGATGTCGTGGATGACGAAGCGCATCCACACGAACCCTGCCTACGCCGCCGCTGTCGGCATGATCGAAGTCCTCAACGAGCCAGTCTCCATGCACGAC
AAGGACAATCGGTATCCTGCCCCTGGTGAGGTACCCGGACTGGTCCAAAAGTACTACCCGGGCGCTCTCAAGGCAGTCCGAGATGCCGAAGCGTCGCTCGGCGTGGCCGAAGGCAACAAGCTCCACGTGCA
ATTCATGTCCCAGAAGTGGGAATCGGGCAACCCGCGGGACAACTCTGCCGTGGCCAACGACAAGCTCAGTGCGTTCGATGACCATAACTACATT

>Cocksfoot (haplotype 2)
ATGATGAGTGACGAGTGGGACAACGTCATGGGTTGCAGCGGGGCTGCCTCCGAGTTCGACTGCATGCTCACCAATTACGGCGGAAGTAAACGAGACGCCGGCAACGAGAAGTTCGAGACTCACTGGAGGACT
TGGATCAACGCCGACAGCGTCCAGTCAGCCCACGACGTTGGCCTGAACACCATTCGCATTCCCATGGGGTACTGGTCCAACGTAGACATCGTCGACAAGGCCAGCGAGCCCTTCGCCGACGGCGACAGGATG
CTCCCCTACCTCGACGCCGTCGTCCAGAAGGCCGCTGACCTTGGCATGTATGTCATCATCGATCTGCACGGGGCACCCGGAGGCCAGCAGGAGGACGTCTTCACCGGCCAGAATAACAAGCCGGCCGGCTTC
TTCAACGACTACTACTTCGGCCGTGCACAGGCGTGGATGTCGTGGATGACGAAGCGCATCCACACGAACCCTGCCTACGCCGCCGCCGTCGGCATGATCGAGGTCCTCAACGAGCCCGTCTCCGGGCACGAC
AAGGACCATCGGTATCCTGCCCCTGGTGAGGTACCCGGACTGGTCCAAAAGTACTACCCGGGCGCTCTCAAGGCAGTCCGAGATGCCGAAGCGTCGCTCGGCGTGGCCGAAGGCAACAAGCTCCACGTGCA
ATTCATGTCCCAGAAGTGGGGCTCGGGCAACCCGCGGGACAACTCTGCCGTGGCCAACGACAAGCTCATTGCGTTCGATGACCACAACTACATT

>Cocksfoot (haplotype 3)
ATGATGAGTGACGAGTGGGACAACGTCATGGGTTGCAGCGGGGCTGCCTCCGAGTTCGACTGCATGCTCACCAATTACGGCGGAAGTAAACAAGACGCCGGCAATGAGAAGTTCGAGACTCACTGGAGGACT
TGGATCAACGCCGATAGCGTCCAGTCAGCCCACGACGTTGGCCTGAATACCATTCGCATTCCCATGGGGTACTGGTCCAACGTAGACATCGTCGACAAGGCCAGCGAGCCCTTCGCCGACGGCGACAGGATG
CTCCCCTACCTGGACGCCGTCGTCCAGAAGGCCGCTGACCTGGGCATGTATGTCATCATCGATCTGCACGGGGCACCCGAAGGCCAGCAGGAGGACGTCTTCACCGGCCAGAACAACAAGCCGGCCGGCTT
CTTCAACGACTACAACTTCGGCCGTGCCCAGGCATGGATGTCGTGGATGACGAAGCGCATCCACACGAACCCTGCCTACGCCGCCGCCGTCGGCATGATCGAGGTCCTCAACGAGCCCGTCTCCGGGCACGA
CAAGGACCATCGGTATCCTGCCCCTGGTGAGGTACCCGGACTGGTCCAAAAGTACTACCCGGGCGCTCTCAAGGCAGTCCGAGATGCCGAAGCGTCGCTCGGCGTGGCCGAAGGCAACAAGCTCCACGTGC
AATTCATGTCCCAGAAGTGGGGCTCGGGCAACCCGCGGGACAACTCTGCCGTGGCCAACGACAAGCTCAGTGCGTTCGATGACCACAACTACATT

>Dactylis marina (haplotype 1)
ATGATGAGTGACGAGTGGAACAACGTCATGGGTTGCAGTGGGGCTGCCTCCGAGTTCGACTGCATGCTCACCAATTACGGCGGAAGTAAACGAGACGCCGGCAACGAGAAGTTCGAGACTCACTGGAGGACT
TGGATCAACGCCGACAGCGTCCAGTCAGCCCACGACGTTGGCCTGAACACCATTCGCATTCCCATGGGGTACTGGTCCAACGTAGACATCGTCGACAAGGCCAGCGAGCCCTTCGCCGACGGCGACAGGATG
CTCCCCTACCTCGACGCCGTCGTCCAGAAGGCCGCTGACCTTGGCATGTATGTCATCATCGATCTGCACGGGGCACCCGAAGGCCAGCAGGAGGACGTCTTCACCGGCCAGAACAACAAGCCAGCCGGCTTC
TTCAACAACTACAACTTCGGCCGTGCCCAGGCATGGATGTCGTGGATGACGAAGCGCATCCACACGAACCCTGCCTACGCCGCCGCCGTCGGCATGATCGAGGTCCTCAACGAGCCCGTCTCCGGGCACGAC
AAGGACAATCGGTATCCTGCCCCTGGTGAGGTACCCGGACTGGTCCAAAAGTACTACCCGGGCGCTCTCAAGGCAGTCCGAGATGCCGAAGCGTCGCTCGGCGTGGCCGATGGCAACAAGCTCCACGTGCAA
TTCATGTCCCAGAAGTGGGAATCGGGCAACCCGCGGGACAACTCTGCCGTGGCCAACGACAAGCTCAGTGCGTTCGATGACCATAACTACATT

>Dactylis marina (haplotype 2)
ATGATGAGTGACGAGTGGAACAACGTCATGGGTTGCAGCGGGGCTGCCTCCGAGTTCGACTGCATGCTCAACAATTACGGCGGAAGTAAACGAGACGCCGGCAACGAGAAGTTCGAGACTCACTGGAGGACT
TGGATCAACGCCGACAGCGTCCAGTCAGCCCACGACGTTGGCCTGAACACCATTCGCATTCCCATGGGGTACTGGTCCAACGTAGACATCGTCGACAAGGCCAGCGAGCCCTTCGCCGACGGCGACAGGATG
CTCCCCTACCTCGACGCCGTCGTCCAGAAGGCCGCTGACCTGGGCATGTATGTCATCATCGATCTGCACGGGGCACCCGGAGGCCAGCAGGAGGACAACTTCACCGGCCAGAATAACAAACCGGCCGGCTTC
TTCAACGACTACAACTTCCGCCGTGCCCGGGCATGGATGTCGTGGATGACGAAGCGCATCCACACGAACCCTGCCTACGCCGCCGCTGTCGGCATGATCGAAGTCCTCAACGAGCCAGTCTCCATGCACGACA
AGGACAATCGGTATCCTGCCCCTGGTGAGGTACCCGGACTGGTCCAAAAGTACTACCCGCGCGCTCTCAAGGCAGTCCGAGATGCCGAAGCGTCGCTCGGCGTGGCCGATGGCAACAAGCTCCACGTGCAAT
TCATGTCCGTGGGGCTCGGGCAACCCGCGGGACAACTCTGCCGTGGCCAACGACAAGCTCAGTGCGTTCGATGACCATAACTACATT

>Dactylis marina (haplotype 3)
ATGATGAGTGACGAGTGGAACAACGTCATGGGTTGCAGCGGGGCTGCCTCCGAGTTCGACTGCATGCTCACCAATTACGGCGGAAGTAAACGAGACGCCGGCAACGAGAAGTTCGAGACTCACTGGAGGACT
TGGATCAACGCCGACAGCGTCAAGTCAGCCCACGACGTTGGCCTGAACACCATTCGCATTCCCATGGGGTACTGGTCCAACGTAGACATCGTCGACAAGGCCAGCGAGCCCTTCGCCGACGGCGACAGGATG
CTCCCCTACCTCGACGCCGTCGTCCAGAAGGCCGCTGACCTGGGCATGTATGTCATCATCGATCTGCACGGGGCACCCGGAGGCCAGCAGGAGGACGTCTTCACCGGCCAGAACAACACGCCGGCCGGCTT
CTTCAACGACTACAACTTCGGCCGTGCCCAGGCATGGATGTCGTGGATGACGAAGCGCATCCACACGAACCCTGCCTACGCCGCCGCTGTCGGCATGATCGAAGTCCTCAACGAGCCAGTCTCCAGGCACGA
CAAGGACAATCGGTATCCTGCCCCTGGTGAGGTACCCGGACTGGTCCAAAAGTACTACCCGGGCGCTCTCAAGGCAGTCCGAGATGCCGAAGCGTCACTCGGCGTGGCCGATGGCAACAAGCTCCACGTGCA
ATTCATGTCCGTGGGAGTCGGGCAACCCGCGGGACAACTCTGCCGTGGCCAACGACAAGCTCAGTGCGTTCGATGACCATAACTACATT

Supplementary Fig. S5

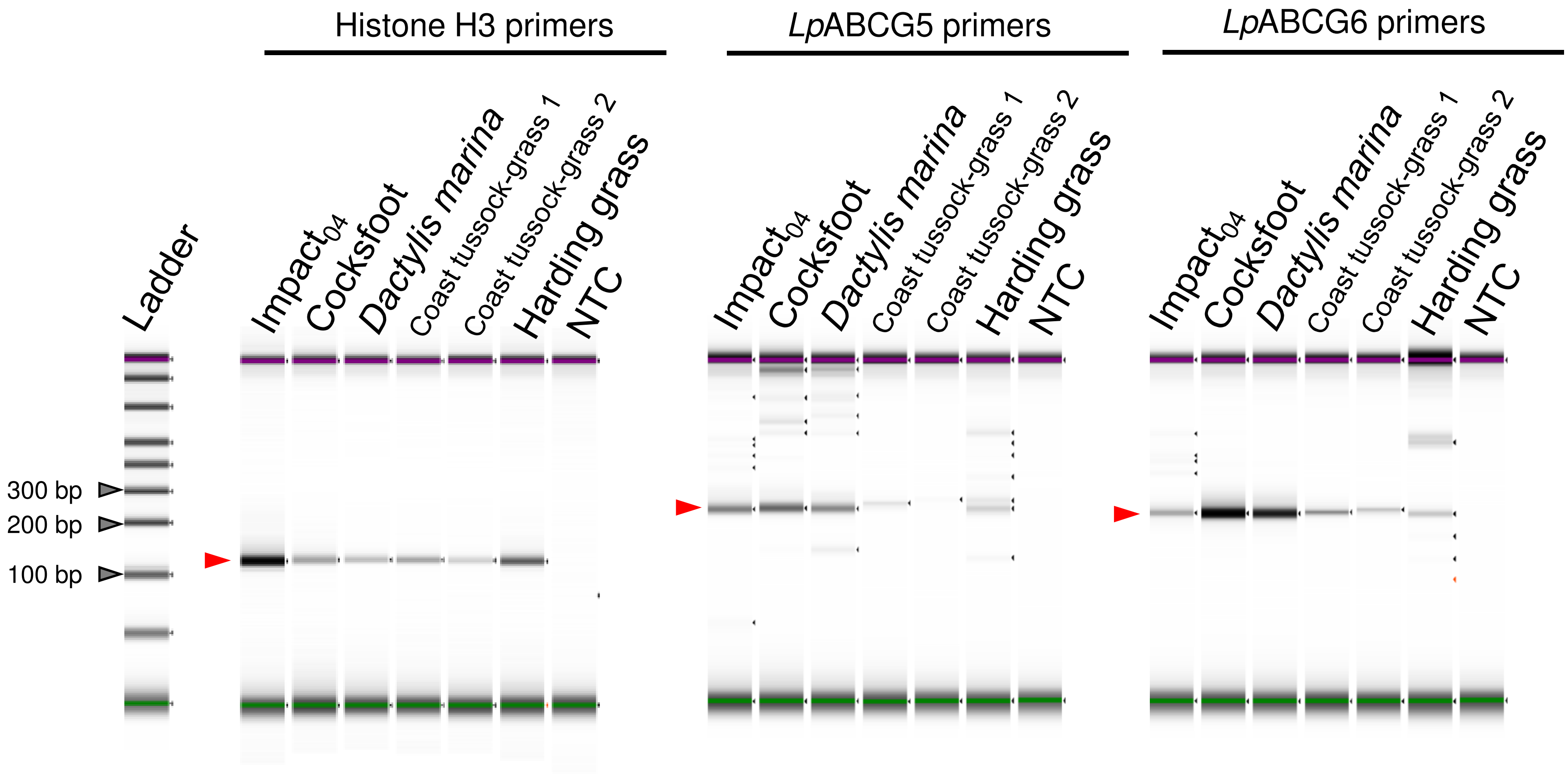

Supplementary Table S1

Species	Sequence identity (length of homologus sequence)				
	NCBI UI	*E. festucae*	*Neotyphodium*	*H. lixii*	*T. harzianum*
Perennial ryegrass	KY771173 (*Lp*BGNL)	90% (1140 bp)	89% (1140 bp)	75% (1101 bp)	74% (1103 bp)
Epichloe festucae	EF015481.1	-	98% (1164 bp)	74% (1101 bp)	73% (1112 bp)
Neotyphodium sp.	AF535131.1	-	-	74% (1283 bp)	73% (1288 bp)
Hypocrea lixii	EU747838	-	-	-	96% (1320 bp)
Trichoderma harzianum	X79197.1	-	-	-	-

Supplementary Table S2

Library	Total reads	Counts	CPM
Root tip	14,656,730	479	32.7
Root (middle)	14,515,287	798	55.0
Leaf middle 1	23,613,687	87	3.7
Leaf middle 2	19,292,712	49	2.5
Leaf middle 3	14,752,797	54	3.7
Leaf tip 1	22,525,848	272	12.1
Leaf tip 2	19,271,284	113	5.9
Leaf tip 3	16,738,932	154	9.2
Flower	11,536,030	767	66.5

Supplementary Table S3

	E⁻ individual					E⁺ individual				
		*Lp*BGNL		*Epichloë* ß-1,6-glucanase gene			*Lp*BGNL		*Epichloë* ß-1,6-glucanase gene	
	Total reads	Counts	CPM	Counts	CPM	Total reads	Counts	CPM	Counts	CPM
0 h	54,109,844	19	0.35	0	0.00	36,389,285	4	0.11	5	0.14
4 h	62,109,304	30	0.48	1	0.02	42,014,889	109	2.59	7	0.17
1 day	74,819,980	2751	36.77	3	0.04	64,295,493	802	12.47	28	0.44
2 days	71,779,944	6285	87.56	1	0.01	57,472,563	2838	49.38	25	0.43
5 days	59,159,973	3391	57.32	1	0.02	57,259,134	1847	32.26	9	0.16
10 days	102,085,102	6565	64.31	0	0.00	53,748,829	3901	72.58	22	0.41

Supplementary Table S4

Species	UI	Instrument	Data size	Submitted by
Dactylis glomerata L. (orchardgrass)	SRX738187	Illumina HiSeq 2000	1 run, 162.8M spots, 32.6G bases	Sichuan Agricultural University
Deschampsia antarctica	SRX465632	Illumina HiSeq 2000	1 run, 153.3M spots, 31G bases	KOREA POLAR RESEARCH INSTITUTE
Poa annua	SRX745831	Illumina HiSeq 2000	1 run, 129.3M spots, 25.9G bases	Auburn University
Poa supina	SRX745855	Illumina HiSeq 2000	1 run, 44.4M spots, 8.9G bases	Auburn University
Poa infirma	SRX745858	Illumina HiSeq 2000	1 run, 46.3M spots, 9.3G bases	Auburn University
Phalaris aquatica	SRX669405	Illumina HiSeq 2000	1 run, 50.4M spots, 10.2G bases	Teagasc

Supplementary Table S5

Primer name	Sequence (5'->3') Forward	Reverse	Amplicon size*	DNA template
LpBGNL_short	GTCGGCATGATTGAGGTTCT	ACTGCACATGGAGCTTGTTG	178 bp	perennial ryegrass, darnel, meadow fescue, tall fescue, sheep fescue, cocksfoot, *Dactylis marina*, coast tussock-grass, *Phalaris aquatica*, perennial ryegrass-associated endophyte, perennial ryegrass genomic library
LpBGNL_indel	AGGGCATCAACAAGATCAGG	CGTCGCTCATCATCCATGGC	253/306 bp	p150/112 genetic mapping population
LpBGNL_long1	CGCGCCTAATCCTCTCCTCT	CAGATATCTTGATACACATTCC	1452 bp	perennial ryegrass, tall fescue, meadow fescue
LpBGNL_long2	GCCCGTCTGACGGGGCACAG	CAGATATCTTGATACACATTCC	1590 bp	darnel
LpBGNL_long3	CATCAACAAGATCAGGGGCG	CTGCCCGTTCACGGTGCGAT	1067 bp	sheep fescue
LpBGNL_long4	CACGACTTGGCTGCTTTCAA	TTTGTCGTCCGGGCTCACGC	1147 bp	cocksfoot, *Dactylis marina*
LpBGNL_cons	CTGCCTCCGAGTTCGACTG	TGGATGCGCYTCGTCATCC	415 bp	perennial ryegrass, cocksfoot, *Dactylis marina*, coast tussock-grass, *Phalaris aquatica*, perennial ryegrass-associated endophyte
LpHistone**	TGCTTGCCCTTCAGGAGGCT	ATCCTCCTGGCAAGCTGAATG	126 bp**	perennial ryegrass, cocksfoot, *Dactylis marina*, coast tussock-grass, *Phalaris aquatica*
LpABCG5	ATCAGGAAGGAGAGCCTCCA	ATGATGGTGTTGGCCGCGTT	248 bp	perennial ryegrass, cocksfoot, *Dactylis marina*, coast tussock-grass, *Phalaris aquatica*
LpABCG6	TAACGCTCAACGGGGACG	TCGTCGCCGATGATGGTGTT	235 bp	perennial ryegrass, cocksfoot, *Dactylis marina*, coast tussock-grass, *Phalaris aquatica*

* Length of DNA fragment based on the perennial ryegrass genome sequence

** Reference: Tu, Y. *et al.* 2010 Functional Analyses of Caffeic Acid O-Methyltransferase and Cinnamoyl-CoA-Reductase Genes from Perennial Ryegrass (*Lolium perenne*). *The Plant Cell* **22**, 3357–3373. (doi:10.1105/tpc.109.072827)

Supplementary Note S1

Figure. Molecular Phylogenetic analysis by Maximum Likelihood method
The evolutionary history was inferred by using the Maximum Likelihood method based on the JTT matrix-based model [1]. The tree with the highest log likelihood (-3092.1463) is shown. The percentage of trees in which the associated taxa clustered together is shown next to the branches. Initial tree(s) for the heuristic search were obtained automatically by applying Neighbor-Join and BioNJ algorithms to a matrix of pairwise distances estimated using a JTT model, and then selecting the topology with superior log likelihood value. The tree is drawn to scale, with branch lengths measured in the number of substitutions per site. The analysis involved 24 amino acid sequences. All positions containing gaps and missing data were eliminated. There were a total of 249 positions in the final dataset. Evolutionary analyses were conducted in MEGA7 [2].

1. Jones D.T., Taylor W.R., and Thornton J.M. (1992). The rapid generation of mutation data matrices from protein sequences. Computer Applications in the Biosciences 8: 275-282.
2. Kumar S., Stecher G., and Tamura K. (2016). MEGA7: Molecular Evolutionary Genetics Analysis version 7.0 for bigger datasets.Molecular Biology and Evolution 33:1870-1874.
